# Fruit Extract Mediated Green Synthesis of Metallic Nanoparticles: A New Avenue in Pomology Applications

**DOI:** 10.3390/ijms21228458

**Published:** 2020-11-11

**Authors:** Harsh Kumar, Kanchan Bhardwaj, Daljeet Singh Dhanjal, Eugenie Nepovimova, Fatih Șen, Hailemeleak Regassa, Reena Singh, Rachna Verma, Vinod Kumar, Dinesh Kumar, Shashi Kant Bhatia, Kamil Kuča

**Affiliations:** 1 Food Technology Department, School of Bioengineering and Food Technology, Shoolini University of Biotechnology and Management Sciences, Solan 173229, Himachal Pradesh, India; microharshs@gmail.com; 2 Botany Department, School of Biological and Environmental Sciences, Shoolini University of Biotechnology and Management Sciences, Solan 173229, Himachal Pradesh, India; kanchankannu1992@gmail.com (K.B.); rachnaverma@shooliniuniversity.com (R.V.); 3 Biotechnology Department, School of Bioengineering and Biosciences, Lovely Professional University, Phagwara 144411, Punjab, India; daljeetdhanjal92@gmail.com (D.S.D.); reena.19408@lpu.co.in (R.S.); 4 Department of Chemistry, Faculty of Science, University of Hradec Kralove, 50003 Hradec Kralove, Czech Republic; eugenie.nepovimova@uhk.cz; 5 Sen Research Group, Biochemistry Department, Faculty of Arts and Science, Dumlupınar University, Evliya Çelebi Campus, 43100 Kütahya, Turkey; fatihsen1980@gmail.com; 6 Biotechnology Department, Applied Sciences and Biotechnology, Shoolini University of Biotechnology and Management Sciences, Solan 173229, Himachal Pradesh, India; haileregg@gmail.com; 7 School of Water, Energy and Environment, Cranfield University, Cranfield MK430AL, UK; Vinod.Kumar@cranfield.ac.uk; 8 Department of Biological Engineering, College of Engineering, Konkuk University, Seoul 05029, Korea; 9 Biomedical Research Center, University Hospital Hradec Kralove, 50005 Hradec Kralove, Czech Republic

**Keywords:** anticancer, antimicrobial, antioxidant, bioactive molecules, catalytic, fruits, metallic nanoparticles

## Abstract

Fruit extracts have natural bioactive molecules that are known to possess significant therapeutic potential. Traditionally, metallic nanoparticles were synthesized via chemical methods, in which the chemical act as the reducing agent. Later, these traditional metallic nanoparticles emerged as the biological risk, which prompted researchers to explore an eco-friendly approach. There are different eco-friendly methods employed for synthesizing these metallic nanoparticles via the usage of microbes and plants, primarily via fruit extract. These explorations have paved the way for using fruit extracts for developing nanoparticles, as they eliminate the usage of reducing and stabilizing agents. Metallic nanoparticles have gained significant attention, and are used for diverse biological applications. The present review discusses the potential activities of phytochemicals, and it intends to summarize the different metallic nanoparticles synthesized using fruit extracts and their associated pharmacological activities like anti-cancerous, antimicrobial, antioxidant and catalytic efficiency.

## 1. Introduction

Nanotechnology has emerged as the revolutionary discipline of science with diverse application in the field of agriculture, biomedicine, catalysis, cosmetics, energy, electronics, mechanics, optics, pharmaceutics, sensors and textile [1]. These diverse applications have led to the advent of advancing discipline like nanobiotechnology [2]. Substantial growth in this field has inclined the interest of researchers to synthesize green nanoparticles (NPs) using different parts of plants. The primary reason for synthesizing metallic nanoparticles from different parts of the plants is that the procedures are cost-effective, eco-friendly, sustainable and straightforward [1,3]. Metal and metal oxide NPs are considered as efficient nanoparticles as they show remarkable biomedical activity and have a high surface area to volume ratio [1]. Predominantly, the bottom-up approach is used for synthesizing biogenic NPs, where atoms and compounds serve as the building block and self-assemble themselves to construct a final product [4]. In this, the biological system performs the function of biological laboratories for synthesizing pure metal as well as metal oxide nanoparticles via a biomimetic approach [5].

Lately, numerous herbal species and plant extracts have been used as capping and reducing agent for synthesizing NPs, which has improved the field of nanoscience [1]. Various bacterial cells, as well as their extracts, have been comprehended for synthesizing varieties of NPs of silver (Ag), gold (Au), silver oxide (AgO), cadmium sulfide (CdS), and titanium dioxide (TiO_2_) [3]. A few fungal species have also been reported for synthesizing NPs of Ag, CdS and TiO_2_. Other biological materials like honey have also been used for NPs from Ag, Au, carbon, lead (Pd) and platinum (Pt) [3]. On extensive analysis, plant-based NPs are more mono-dispersed and stable in contrast to microbial NPs.

Additionally, plants extracts have added advantage as they take less time to reduce the metal ions. The main reason for considering the natural plant-based extracts for synthesizing NPs is the use of green chemistry in their synthesis. The primary benefit of using green chemistry for synthesizing NPs is that it allows the selection of an eco-friendly reducing agent, solvent medium and non-toxic material for stabilization of NPs [3]. Diverse compounds like alkaloids, amines, amides, flavanones, terpenoids, proteins, phenolics and pigments are present in the plant extract, which aid in reduction and stabilization of metal ions during green NP synthesis [6]. Moreover, compounds like sugars, vitamins, peptides and water from tea and coffee extracts have been effectively used for synthesizing NPs [7,8,9,10,11,12]. Therefore, the current review intends to highlight the use of fruit extracts for synthesizing green metallic NPs. The first portion of the review discusses the role of fruits in human life and the second portion of the review emphasizes the different metallic NPs obtained through various fruit extracts as well as their applications.

## 2. Health Benefits of Fruit Phytochemicals

**Figure 1:**
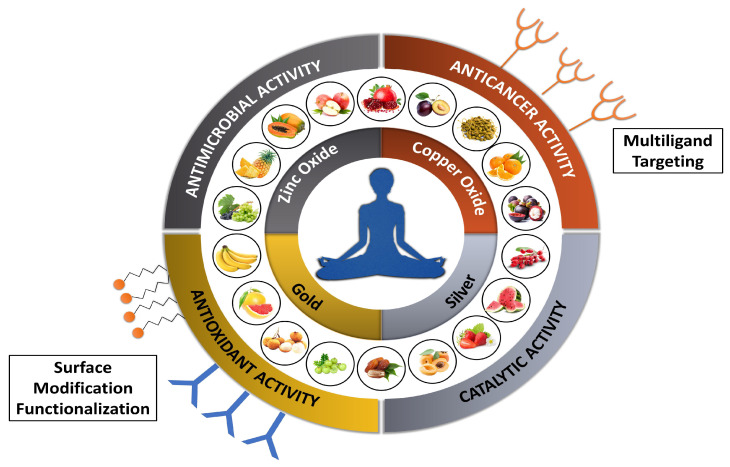
Graphical representation of fruit sources used for synthesizing nanoparticles with potential biological activities.

The ingestion of fruit and its products not only improves the health of the individual but also reduces the chances of different diseases like age-related macular degeneration, ageing, cardiovascular disease, cancer, cataract of the eye, compromised immune system, gastrointestinal disorder, hypertension, high cholesterol and lowering of low-density lipoprotein (LDL) [13]. To endorse a healthy lifestyle, USDA (United States Department of Agriculture) has recommended filling half of the plate with vegetables and fruits. This is because they have a substantial amount of dietary fibre, minerals (calcium, iron, magnesium and potassium), vitamins (ascorbic acid, folic acid, as well as vitamin A precursors) and various beneficial phytochemicals with antioxidant properties. Lately, FAO/WHO (Food and Agriculture Organization of the United Nations and World Health Organization) have stated to consume minimum 400g of vegetable and fruits daily (exclusive of potatoes as well as other starchy tubers) to prevent chronic diseases (cancer, diabetes, heart disease and obesity) and alleviate the level of deficient micronutrients [14]. Origin and temperature of production area are the key factors which further enable the classification of fruits into tropical fruits, sub-tropical fruits and temperate fruits [13]. The NPs obtained from fruits has been known to possess various biological activities, as depicted in Figure 1.

Carotenoids are the plant pigments that provide the red, yellow and orange colour to fruits. Approximately 600 carotenoids have been identified to date, out of which, around 50 get transformed into vitamin A [15]. Other than this, carotenoids are also known for their antioxidant potential and claimed for reducing the risk of diseases like cancer, cataracts, cardiovascular and macular degeneration. Additionally, they have a substantial role in improving the immune system [16,17]. Orange coloured fruits are the primary reservoir of β-carotene that are known for exhibiting highest provitamin A activity. Red carotenoid, i.e., lycopene, is predominantly found in pink grapefruit, tomato, watermelon and other red-coloured fruits and has been acclaimed as the valuable antioxidant in carotenoids family. Various animal, as well as human studies, have revealed that lycopene shows defending nature against different carcinogens of breast, brain, cervix, colon and prostate [18,19,20,21].

Furthermore, flavonoids are a group of phenolic compounds encompassing anthocyanins, flavanones, catechins, flavonols, flavones and isoflavones. To date, approximately 4000 flavonoids have been identified, and these flavonoids are dominantly found in citrus fruits and berries [15]. Published literature related to flavonoids has revealed that they have a myriad of benefits on humans like the potential to prevent cardiovascular disorders, cancers, urinary tract infections (UTIs) and other degenerative diseases [22,23,24]. Flavonoid Anthocyanins are blue pigments in blueberry and red pigments in strawberry and cherry. In addition to that, anthocyanins have been claimed to show to antioxidant potential in a biological system. Blackberry, Blueberry, Black raspberry and cranberry contain flavonoid proanthocyanidins, which have a substantial role in reducing the chances of UTIs [15]. The different phytochemicals obtained from fruits are used as a capping agent in NPs synthesis, and their biological applications in humans have been illustrated in Table 1.

## 3. Green Synthesis of Nanoparticles Mediated by Fruit Extracts

**Figure 2:**
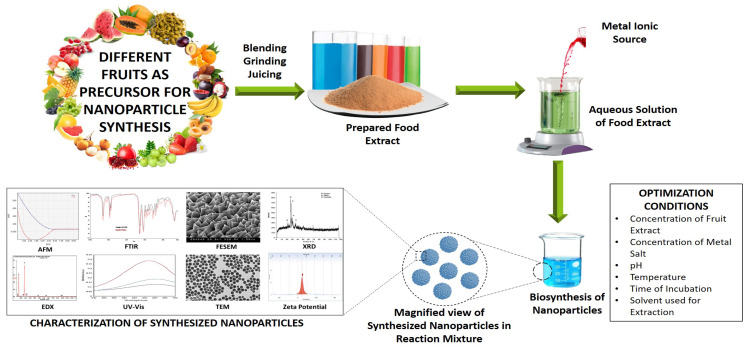
A general mechanism of nanoparticle formation from fruit extracts.

Extracts of fruits have been comprehended to contain a high amount of reducing agents. For example, fruits like blueberries, blackberries, *Cornus mas* L., *Citrullus lanatus*, grape, *Terminalia arjuna* and *Punica granatum* L., comprise a high number of anthocyanins, ascorbic acid, phenolic compounds, flavonoids, saccharides and other vitamins [53]. The synthesis of NPs from fruits has an additional advantage in comparison to NPs synthesized by the biological method. The biological method for NP synthesis is mediated by microbes, and microbes need to be pure strains and ought to be maintained in an aseptic environment. Moreover, the separation of nanoparticles from microbial broth culture during downstream processing is quite tricky. Furthermore, it takes time to transform the metallic salts (soluble) into the elemental oxide/elemental NPs [3]. A general mechanism involved in the biosynthesis of diverse nanoparticles using fruit extracts has been illustrated in Figure 2.

The data related to the synthesis of NPs using different varieties have been comprehended in Table 2.

The diverse types of nanoparticles prepared using different fruits extracts have been discussed below: 

### 3.1. Copper Oxide Nanoparticles (Cu_2_ONPs)

Copper oxide (Cu_2_O) is claimed to be a transition metal oxide having narrow bandgap, i.e., ~2.0 eV and shows distinct features like significant electrochemical activity, improved redox potential, high specific surface area and incomparable stability in solutions. It is the second choice of researchers working in the field of nanotechnology after the noble metal nanoparticles, owing to its propitious application in different subjects like antifouling coatings, biocidal agents, catalysis, sensors/biosensors, electrochemistry and energy storage [72]. These nanoparticles are extensively used for non-enzymatic sensing of clinical analytes due to their ability to promote electron transfer reaction even at low potential [72]. The Cu_2_ONPs derived from fruits have been listed along with various applications in Table 3.

### 3.2. Gold Nanoparticles (AuNPs)

In recent years, Gold nanoparticles (AuNPs) have gained substantial attention owing to their biocompatibility, optical and physical (shape and size) properties [73]. AuNPs of diverse morphology and varied size are extensively employed in medicine for different purposes such as for the detection of tumours, as a drug carrier, etc. [73]. The AuNPs derived from fruits has been listed along with various applications in Table 4.

### 3.3. Silver Nanoparticles (AgNPs)

Silver nanoparticles (AgNPs) have also gained significant attention due to their biochemical and catalytic activity owing to their large surface area in contrast to other particles with analogous chemical structures [73]. Synthesis of AgNPs occurs in two steps: in the first step, Ag^+^ ions are reduced to Ag^°,^ and in the second step, clustering of colloidal AgNPs take place to form oligomeric clusters which finally gets stabilized [73]. The reduction of Ag^+^ ions requires biological catalysts, i.e., enzymes, which are obtained from different biological sources like microbes, fruits extracts, plants, etc. Moreover, a variety of fruits extracts have already been comprehended for synthesizing AgNPs with diverse biological potential, as listed in Table 5.

### 3.4. Zinc Oxide Nanoparticles (ZnONPs)

Lately, ZnONPs has intrigued researchers working in the field of nanotechnology owing to their diverse application in different areas such as biomedical, electronics and optical sector [76]. Synthesis of ZnONPs is considered to be cost-effective, easy and safe. Even, FDA has given the generally recognized as safe (GRAS) status to ZnO [77,78]. ZnONPs have been primarily comprehended for anti-inflammatory properties and wound healing in the medical sector [76]. Nowadays, ZnONPs are predominantly used in cosmetic products like sunscreen lotions, as they exhibit intrinsic UV filtering potential [76]. Other than this, ZnONPs are used in drug delivery system as they show anti-cancerous, antifungal, antimicrobial and anti-diabetic properties [76]. The ZnONPs derived from fruits has been listed along with various applications in Table 6.

## 4. Anticancer Activity of Fruit-Derived NPs

Cancer is one of the leading diseases, which has accounted for 9.6 million deaths worldwide in 2018, where 70% of deaths occurred in low- and middle-income countries [79]. Chemotherapy, hormone therapy, radiation therapy and surgery are the primary approaches applied for management and treatment of cancer. Various medicinal plants having anti-cancerous and cytotoxic potential have already been recorded [80]. Polyphenols like alkaloids, flavonoids, phenolic acids and terpenes have been claimed to be responsible for the biological activity of plants [81,82,83]. Triterpenoids like avicins, boswellic acids, fomitellic acids, oleanolic acid, pomolic acid and ursolic acid have been comprehended for exhibiting cytotoxic effects [84]. Flavonoids like rutin, myricetin, kaempferol and quercetin have also been reported to show anticancer potential [82]. Application of nanotechnology in combating cancer has unfolded the new avenues for interdisciplinary research involving the different fields like biology, chemistry, engineering and medicine for diagnosis, detection and treatment [85]. In recent times, Myocet™ (Perrigo, Dublin, Ireland), Doxil^®^ (Johnson and Johnson, New Brunswick, NJ, USA) and Abraxane^®^ (Celgene, Summit, NJ, USA), the nano-based anti-cancerous drugs have been approved for clinical use by Food and Drug Administration (FDA-USA) [86].

AuNPs have been prepared using *Citrus macroptera* (CM) juice and have been evaluated for anti-cancerous potential via in vitro analysis on HepG2 liver cancer cells [55]. The result obtained from the study showed the IC_50_ value of 70.2 ng/mL for CM-AuNPs. This study was conducted to unveil the real potential of CM as it is used in traditional medicine by Tripura tribal people for treating liver ailments. Khan et al. [36] developed AuNPs using longan fruit juice and evaluate for anti-cancerous activity on MCF-7 (human breast cancer cells). The result obtained for the study revealed that increasing the concentration of AuNPs to 6.25–100 μg/mL substantially decreases the viability of these cells [36]. Moreover, spherical morphology, small size (25 nm), capping of phytochemicals and uniform distribution are considered as critical factors to exhibit considerable anti-cancerous potential. Additionally, the capping phytochemicals also help in regulating the expression of pro-apoptotic Bax and anti-apoptotic Bcl-2 protein

Jacob et al. [45] developed AgNPs via dried fruit extract of *Ficus carica* and evaluated for cytotoxic potential against MCF-7 (human breast cancer cells). The result showed the LD_50_ of AgNPs was 12.411 μg in contrast to fruit extract, which was 139.04 μg. The analysis of cytotoxic potential was done assessing the level of ROS. On the other hand, *Phoenix dactylifera* mediated AgNPs exhibited dose-dependent cytotoxicity against MCF-7 [58]. The result obtained from the study showed the highest inhibitory effect at a concentration of 200 μg/mL. *Ananas comosus* (L.) peel extract mediated AgNPs have also been recorded for anti-cancerous activity against HepG2 cancer cell line at high concentrations [67]. AgNPs derived from peel extract of *Punica granatum* have been reported to show 55–62% toxicity at a dosage of 12.5 μg against cell line of colon cancer [66]. The study enlightens us about the programmed cell death through autophagy. AgNPs prepared using peel extract of *Dimocarpus Longan Lour* have also been reported to show dose-dependent cytotoxic effects against prostate cancer cell line (PC-3) [68]. The concentration of AgNPs in the range of 5–10 μg/mL was found to reduce the viability of PC-3 cells by 50%. Moreover, the IC_50_ was also less than 10 μg/mL and cytotoxic potential of these NPs was confirmed by suppression in the expression of BCL-2, STAT3 and survivin, whereas upregulation of the expression of caspase-3. Furthermore, AuNPs derived using grape peel and seeds have been evaluated for cytotoxic potential. The action of AuNPs caused the increase in the level of ROS, stimulated apoptosis as well as apoptotic morphological changes in A431 cells. These changes were claimed to be associated with interference of AuNPs with mitochondrial membrane potential [74,75]. It was concluded in the study that the reduction in mitochondrial membrane potential triggers the apoptotic cascade reaction in AuNPs treated cells. 

## 5. Antimicrobial Activity of Fruit-Derived NPs

NPs have the potential to show antimicrobial activity, as NPs pass via the membrane of bacteria which influence the cell activity along with metabolic pathways [87]. After entering and interfering with the metabolic pathway of the bacteria cell, NPs adhere themselves with elementary components such as DNA, enzymes, liposomes and ribosomes. The association of elementary components with NPs leads to heterogeneous alterations, oxidative stress and alterations in cell membrane permeability, deactivation of proteins, enzyme inhibition, imbalance of electrolytes and alterations in gene expression [87].

Cell membranes and walls serve as protective checkpoints and impart resistance to the bacteria from the external environment. The bacterial cell wall also plays a primary role in conferring shape to the bacteria. Diverse pathways are involved in the absorption of NPs via the cell membrane in both Gram-positive and Gram-negative bacteria [88]. In Gram-negative bacteria, lipopolysaccharides (LPS) offer a negative charge for attracting NPs. Whereas, teichoic acid is present in Gram-positive bacteria to perform a similar function. Hence, NPs circulate through the phosphate molecular chain and circumvent accumulation. Moreover, NPs are found to be effective against Gram-positive in contrast to Gram-negative, owing to the presence of lipoproteins, LPS and phospholipids, which acts as the barrier and allows the movement of macromolecules only. Whereas, cell death and damage to cell membrane takes place in Gram-positive bacteria as the cell wall comprises teichoic acid, a thin layer of peptidoglycan and has abundant pores which allow the entry of foreign molecules [87].

Cu_2_ONPs derived by using *Ziziphus spina-christi* (L.) extract has been reported for exhibiting antimicrobial activity against *Staphylococcus aureus* in contrast to *Escherichia coli* [28]. Another study reported about Cu_2_ONPs derived from fruit extract of *Capparis spinosa*, which showed resilient antimicrobial activity against *Bacillus cereus* and *S. aureus* in contrast to *E. coli* and *Klebsiella pneumoniae* [29]. *Citrus macroptera* fruit extract mediated AuNPs have also been reported for inhibiting the proliferation of biofilm-producing *Pseudomonas aeruginosa*. The result from the study revealed that 12 ng/mL concentration of particles was effective for inhibiting the 60% growth of the biofilm [55]. Extract of *Emblica officinalis* has been used for synthesizing AgNPs. The synthesized AgNPs have been reported to exhibit high antimicrobial potential in contrast to fruit extract *E. officinalis* alone [43]. Biological derived AgNPs have been recorded to have effective antimicrobial activity against *Bacillus subtilis, E. coli, K. pneumoniae* and *S. aureus* [43]. AgNPs derived using fruit extract of *Crataegus pentagyna* has been accorded with minimum inhibitory concentration and minimum bactericidal concentration of 0.11, 0.22, 0.11, 0.44, 0.11, 1.7, 0.11, 0.22 and 0.11, 7.1 μg/mL against *Acinetobacter baumannii, E. coli, Enterococcus faecalis*, *P. aeruginosa* and *S. aureus*, respectively [47]. Furthermore, ZnONPs derived using an extract of *Citrus maxima* has been recorded to exhibit significant antimicrobial activity against pathogenic microbes like *Klebsiella aerogenes* and *S. aureus*, whereas, and less significant towards *E. coli* [69].

## 6. Antioxidant Activity of Fruit-Derived NPs

Cu_2_ONPs derived using strawberry has been reported to exhibit remarkable concentration-dependent DPPH radical scavenging activity [30]. The interaction of strawberry extract with both Cu_2_ONPs and DPPH occurs via the transfer of electrons as well as hydrogen ions to 2,2-diphenyl-1-picrylhydrazyl radical to convert itself to a 2,2-diphenyl-1-picrylhydrazine molecule (DPPH). Usually, DPPH shows strong absorbance at a wavelength of 517 nm. However, due to the gain of an electron/hydrogen atom from an antioxidant molecule, it transforms and forms a steady diamagnetic molecule which showed a reduction in absorbance at 517 nm. Additionally, the transformation of colour from purple to pale yellow helps to determine the anti-radical potential of antioxidants [30]. Anthocyanins, flavanols, flavan-3-ols and tannins (ellagitannins and gallotannins) are some of the antioxidant molecules obtained from strawberry [30]. These compounds have been comprehended for maintaining the redox homeostasis via following multiple steps of antioxidant reactions which includes initiation, branching, propagation and termination steps of free radicals.

AuNPs derived from the juice of Longan fruit has been accorded to show dose-dependent antioxidant activity against DPPH, which increases with increase in AuNPs [36]. Gubitosa et al. [37] conducted the study to assess the antioxidant potential of AuNPs derived using *Punica granatum* juice on H_2_O_2_ (ROS model). Protein cytochrome-c is highly sensitive to H_2_O_2,_ but oxidative degradation alters its catalytic potential. The result obtained from the study unveils that an increase in AuNPs significantly decreases the Cyt-c degradation rate. In simple words, the presence of AuNPs delays the Cyt-c degradation process. AgNPs synthesized by Cavendish banana peel extract (CBPE) has been evaluated for antioxidant potential against DPPH [63]. The result obtained revealed that the inhibition rate of CBPE-mediated AgNPs is 64% which increases with an increase in AgNP concentration. Moreover, CBPE-mediated AgNPs have also been reported for scavenging 2,2′-azinobis-(3-ethylbenzothiazoline-6-sulfonic acid) (ABTS).

## 7. Catalytic Activity of Fruit-Derived NPs

The compound 4-nitrophenol and its derivatives are predominantly found in synthetic dyes, herbicides and insecticides, which are chiefly organic pollutants accorded from harming ecosystems [89]. Due to the inhibitory and toxic nature of 4-nitrophenol, it is regarded as a massive risk to the ecosystem. Hence, the degradation of these pollutants has become a matter of utmost importance. Despite this, the reduced product of 4-nitrophenol is used as a mediator in black/white film developers, rubber antioxidants, sulfur dyes, paracetamol, corrosion inhibition and act as precursors in analgesic and antipyretic drugs [90,91]. NaBH_4_ is extensively used as a metal catalyst, and reductant for Cu_2_ONPs, AgNPs, AuNPs and PdNPs; it is claimed to be the most effective method to reduce 4-nitrophenol [92,93,94,95]. Methylene blue (MB) is a heterocyclic aromatic pollutant released from the dying industries [96]. NaBH_4_ is used as a reducing agent for MB and NPs, which serves the purpose of an absorbent [97]. 

Cu_2_ONPs derived using fruit extract of *Ziziphus spina-christi* (L.) has been reported to absorb 95% of crystal violet under controlled conditions, i.e., pH 9, the dye concentration of 35 μg mL^−1^, stirring time of 7.5 min and sorbent amount of 80 mg [28]. Colloidal gold nanoparticles synthesized using juice extract of *Punica granatum* have been reported to reduce 4-nitrophenol to 4-aminophenol in 12 min [35]. Whereas, AuNPs derived using *Citrus maxima* took 22 min for the same [38]. *Terminalia chebula* derived AgNPs have been reported for maximum reduction of MB to leucomethylene blue (colourless) within 30 min via electron relay effect [42]. Ebrahimzadeh et al. [47] reported the degradation of organic dyes like eosin (EY), methylene blue (MB) and rhodamine b (RhB) with degradation percentages of 70%, 78% and 85%, respectively, by *Crataegus pentagyna* fruit extract-derived AgNPs within 90 min under sunlight. ZnONPs derived using fruit pericarp of *Garcinia mangostana* have been reported to degrade malachite green dye by 99% within 180 min under sunlight in aqueous solution due to effective oxidation via hydroxyl radicals (^•^OH) synthesized during photocatalytic reactions [52].

## 8. Conclusions

Nature has its unique way of creating highly efficient miniature functionalized materials. Increased responsiveness in the direction of green chemistry and its use in synthesizing metallic nanoparticles has generated an aspiration to develop eco-friendly approaches. The advantage of using fruit extracts for synthesizing nanoparticles is that they are cost-effective, economical, energy-efficient, safe, and environment-friendly, do not affect human health and produce less waste. These green synthesized nanoparticles are being evaluated in the field of nanotechnology for diverse applications. Moreover, the use of fruit extracts for synthesizing nanoparticles has the added advantage over other biological procedures, which are time-consuming and require the maintenance of microbial cultures to sustain the actual potency during the nanoparticle synthesis. Therefore, the usage of plant extracts for deriving nanoparticles can have a massive impact in the coming years. Several works can be cited for the synthesis of nanoparticles via fruit extracts. But still, there is a substantial need for an economic, commercially viable and eco-friendly approach, which explores the potential of natural reducing agents to synthesize nanoparticles that is under exploration. Moreover, there is substantial variation in the chemical composition of fruit extracts of the same species of fruits procured from different areas of the world and which could provide us with varying results in different laboratories. Therefore, exploration of biomolecules playing a significant role in nanoparticles synthesis has become an emerging field and an uncovered avenue for research.

## Figures and Tables

**Table 1:** Enlist of phytochemicals obtained from fruits with their role as a capping agent in metallic nanoparticles for human benefits.

Fruit Verities	Phytochemicals	Role	Types of Metallic Nanoparticles	Phytochemicals as Capping Agents	References
Banana, Amla, Pomegranate	Trans-β carotene, β-Sitosterol, Caffeic acid, Gallagic acid	Anticancer	Copper oxide	Phenols, Primary amines, Polyphenols, Sterols, Fatty acids, Hydroxyl, Carbonyl, Terpenoids, Proteins	[13,25,26,27,28,29,30,31,32]
Banana, Amla, Pomegranate, Guava, Citron	p-Coumaric acid, Vitamin C, Emblicanin-A, Catechin, Guavin-B, β-Bisabolene	Antioxidants	Gold	Carboxyl, Aliphatic amines, Phenols, Flavonoids, Terpenes, Vitamins, Lycopene, Glycosides, Amino acids	[13,25,26,33,34,35,36,37,38,39,40]
Banana, Amla, Guava, Citron	Ferulic acid, 1,6-bis-O-galloyl-beta-d-glucose, Gallic acid, Avicularin, Citral B, Limonene,	Antimicrobial	Silver	Phenols, Alkaloids, Vitamins, Polyphenols, Amino acids, Carbohydrates, Proteins, Flavonoids	[13,25,33,34,41,42,43,44,45,46,47]
Pomegranate, Lemon, Grape, Pineapple, Jamun	Punicic acid, Β-pinene, Stilbenoid, Malic acid, Bromelain	Skincare	Zinc oxide	Phenols, Flavonoids, Xanthones, Anthocyanin	[26,48,49,50,51,52]

**Table 2:** Synthesis of nanoparticles (NPs) from various fruit verities.

Fruit Common Name	Scientific Name	Biological Extract	Types of NPs synthesized	Reaction Temperature/Time	Morphology	Size	Stability	References
North Arcot	*Syzygium alternifolium* (Wt.) Walp	Whole fruit	Copper oxide	50 °C/2 h	Sphere	2–69 nm	Nd	[27]
Christ’s thorn jujube	*Ziziphus spina-christi* (L.)Willd	Pulp	Copper oxide	80 °C/ND	Sphere	5–20 nm	Nd	[28]
Caperberry	*Capparis spinosa*	Whole fruit	Copper oxide	60 °C/24 h	Sphere	17–41 nm	Nd	[29]
Citron	*Citrus medica* Linn.	Juice	Copper oxide	60–100 °C/ND	ND	10–60 nm	Nd	[54]
Strawberry	*Fragaria ananassa*	Whole fruit	Copper oxide	RT/1 h	Sphere	10–30 nm	Nd	[30]
Guava	*Psidium guajava* L	Whole fruit	Copper oxide	80 °C/2 h	Flakes	15–30 nm	15 days	[31]
Pomegranate	*Punica granatum*	Peel	Copper oxide	80 °C/10 min	Sphere	15–20 nm	Nd	[32]
Pomegranate	*Punica granatum*	Juice	Gold	RT/ND	Triangular, Pentagonal, Hexagonal and Sphere	23–36 nm	Nd	[35]
Wild orange	*Citrus macroptera*	Juice	Gold	40–50 °C/90 min	Sphere	7–25 nm	Nd	[55]
Longan	*Euphoria longana* Lam.	Juice	Gold	30 °C/30 min	Sphere	25 nm	Nd	[36]
Pomegranate	*Punica granatum*	Juice	Gold	RT/20 min	Irregular	100 nm	Nd	[37]
Pomelo	*Citrus maxima*	Juice	Gold	RT/5 min	Rod and Sphere	25.7 nm	Nd	[38]
Watermelon	*Citrullus lanatus*	Rind	Gold	RT/1 h	Sphere	20–140 nm	1 month	[39]
Plum	*Prunus domestica*	Whole fruit	Gold	RT/4 h	Sphere	4–38 nm	Nd	[40]
Pomegranate	*Punica granatum*	Juice	Silver	65 °C/1 min	Cubic	23 nm	Nd	[56]
Papaya	*Carica papaya*	Juice	Silver	NS	Sphere	75.68 nm	Nd	[41]
Chebulic myrobalan	*Terminalia chebula*	Whole fruit	Silver	RT/ND	Cubic	25 nm	Nd	[42]
Grape	*Vitis vinifera*	Whole fruit	Silver	RT/4 h	Sphere	30–40 nm	Nd	[57]
Indian gooseberry	*Emblica officinalis*	Pulp	Silver	RT/30 min	Sphere	15 nm	Nd	[43]
Indian gooseberry	*Phyllanthus emblica*	Pulp	Silver	65 °C/20 min	Sphere	19.8–92.8 nm	Nd	[44]
Fig	*Ficus carica*	Whole fruit	Silver	RT/24 h	Sphere	54–89 nm	Nd	[45]
Indian gooseberry	*Phyllanthus emblica*	Pulp	Silver	RT/ND	Cubic	19–45 nm	Nd	[46]
Black hawthorn	*Crataegus pentagyna*	Pulp	Silver	RT/2 h	Sphere	25–45 nm	Nd	[47]
Date palm	*Phoenix dactylifera*	Pulp	Silver	60 °C/20 min	Sphere	20–100 nm	Nd	[58]
Date palm	*Phoenix dactylifera*	Pulp	Silver	55 °C/10 min	Sphere	25–60 nm	Nd	[59]
Apple	*Malus pumila*	Pulp	Silver	80 °C/ND	Sphere	30.25 nm	Nd	[60]
Pomegranate	*Punica granatum*	Peel	Silver	RT/24 h	ND	5–50 nm	Nd	[61]
Banana	*Musa paradisiaca*	Peel	Silver	30 °C/ND	Sphere	23.7 nm	Nd	[62]
Banana	*Musa paradisiaca*	Peel	Silver	RT/30 min	Grain	34 nm	Nd	[63]
Orange	*Citrus sinensis*	Peel	Silver	90 °C/15 min	Sphere	7.36 nm	Nd	[64]
Apricot	*Prunus armeniaca*	Peel	Silver	NS	Rod	50 nm	Nd	[65]
Pomegranate	*Punica granatum*	Peel	Silver	RT/24 h	Sphere	20–40 nm	Nd	[66]
Pineapple	*Ananas comosus*	Peel	Silver	RT/24 h	Sphere	ND	Nd	[67]
Logan	*Dimocarpus Longan Lour*	Peel	Silver	80 °C/5 h	Cubic	9–32 nm	6 months	[68]
Pomelo	*Citrus maxima*	Juice	Zinc oxide	400 °C/5–10 min	Agglomerated	10–20 nm	Nd	[69]
Purple mangosteen	*Garcinia mangostana*	Pulp	Zinc oxide	70–80 °C/ND	Sphere	21 nm	Nd	[52]
Pomegranate	*Punica granatum*	Peel	Zinc oxide	80 °C/ND	Sphere and Hexagonal	32–81 nm	Nd	[70]
Pineapple	*Ananas comosus*	Juice	Zinc oxide	240 °C/5 min	ND	30–57 nm	Nd	[71]

RT—room temperature; ND—not defined; NS—not specified; Nd—not determined.

**Table 3:** Applications of copper oxide NPs synthesized from various fruit varieties.

Family	Fruit Verity	Applications	References
Myrtaceae	*Syzygium alternifolium* (Wt.) Walp.	Antiviral activity against Newcastle Disease Virus (NDV)	[27]
Rhamnaceae	*Ziziphus spina-christi* (L.)Willd	Adequate adsorption capacity to the removal of crystal violet (CV), from aqueous solution; Antibacterial activity against *Escherichia coli* and *Staphylococcus aureus*	[28]
Capparaceae	*Capparis spinosa*	Antibacterial activity against *S. aureus*, *Bacillus cereus*	[29]
Rutaceae	*Citrus medica* Linn.	Antibacterial activity against *E. coli*, *Klebsiella pneumoniae*, *Pseudomonas aeruginosa*, *Propionibacterium acnes* and *Salmonella typhi*; Antifungal activity against *Fusarium culmorum*, *F. oxysporum* and *F. graminearum*	[54]
Rosaceae	*Fragaria ananassa*	Antibacterial activity against *S. aureus*, *S. saprophyticus*, *Bacillus subtilis*, *Streptococcus pneumoniae*, *E. coli* O157: H7, *S. typhimurium*, *Proteus mirabilis*, and *P. aeruginosa*; Antifungal activity against *Candida guilliermondii*, *C. parapsilosis*, *C. albicans*, *C. krusei*, and *C. glabrata*; Antioxidant activity; Cutaneous wound healing ability	[30]
Myrtaceae	*Psidium guajava* L	Antibacterial activity against *E. coli* and *S. aureus*	[31]
Lythraceae	*Punica granatum*	Antibacterial activity against *Micrococcus luteus* MTCC 1809, *P. aeruginosa* MTCC 424, *Salmonella enterica* MTCC 1253 and *Enterobacter aerogenes* MTCC 2823	[32]

**Table 4:** Applications of gold NPs synthesized from various fruit varieties.

Family	Fruit Verity	Applications	References
Lythraceae	*Punica granatum*	Catalytic activity against 4-nitrophenol	[35]
Rutaceae	*Citrus macroptera*	Antibiofilm activity against *Pseudomonas aeruginosa*; Cytotoxic effect against HepG2 (liver cancer cell line)	[55]
Sapindaceae	*Euphoria longana* Lam.	Cytotoxicity against human breast cancer cell lines MCF-7; Antioxidant activity	[36]
Lythraceae	*Punica granatum*	Antioxidant activity	[37]
Rutaceae	*Citrus maxima*	Catalytic activity against 4-nitrophenol	[38]
Cucurbitaceae	*Citrullus lanatus*	Antibacterial activity against *Bacillus cereus* ATCC 13061, *Escherichia coli* ATCC 43890, *Listeria monocytogenes* ATCC 19115, *Staphylococcus aureus* ATCC 49444, *Salmonella typhimurium* ATCC 43174; Antioxidant activity; Anti-proteasome inhibitory potential	[39]
Rosaceae	*Prunus domestica*	Catalytic activity against 4-nitrophenol	[40]
Vitaceae	*Vtis vinifera*	Apoptotic activity against human epidermoid carcinoma A431 cell line	[74,75]

**Table 5:** Applications of silver NPs synthesized from various fruit varieties.

Family	Fruit Verity	Applications	References
Combretaceae	*Terminalia chebula*	Catalytic activity against methylene blue	[42]
Vitaceae	*Vitis vinifera*	Antibacterial activity against *Bacillus subtilis* and *Klebsiella planticola*	[57]
Phyllanthaceae	*Emblica officinalis*	Antibacterial activity against *Staphylococcus aureus*, *B. subtilis*, *Escherichia coli* and *Klebsiella pneumoniae*	[43]
Phyllanthaceae	*Phyllanthus emblica*	Antibacterial activity against *Acidovorax oryzae* strain RS-2	[44]
Moraceae	*Ficus carica*	Cytotoxicity against human breast cancer cell lines MCF-7	[45]
Phyllanthaceae	*Phyllanthus emblica*	Antibacterial activity against *S. aureus*, *K. pneumoniae*	[46]
Rosaceae	*Crataegus pentagyna*	Antibacterial activity against *E. coli*, *S. aureus*, *Enterococcus faecalis*, *Pseudomonas aeruginosa*, *Acinetobacter baumannii*; Photocatalytic action against rhodamine b, eosin and methylene blue	[47]
Arecaceae	*Phoenix dactylifera*	Antibacterial activity against *E. coli*, *S. aureus*, *E. faecalis*, *P. aeruginosa*; Antifungal activity against *Candida albicans*; Cytotoxicity against human breast cancer cell lines MCF-7	[58]
Arecaceae	*Phoenix dactylifera*	Antibacterial activity against *S. aureus*, *S. epidermidis*, *K. pneumoniae*, and *E. coli*; Catalytic activity against 4-nitrophenol	[59]
Rosaceae	*Malus pumila*	Antibacterial activity against *E. coli*, *S. aureus*, *P. aeruginosa* and methicillin-resistant *S. aureus*	[60]
Lythraceae	*Punica granatum*	Antibacterial activity against *E. coli*, *S. aureus*, *P. aeruginosa*	[61]
Musaceae	*Musa paradisiaca*	Antibacterial activity against *E. coli*, *S. aureus*, *P. aeruginosa*, *B. subtilis*; Antifungal activity against *Candida albicans*	[62]
Musaceae	*Musa paradisiaca*	Antibacterial activity against *E. coli*, *K. pneumoniae*, *S. aureus*, *B. subtilis*; Antioxidant activity	[63]
Rosaceae	*Prunus armeniaca*	Antibacterial activity against *E. coli*, *S. aureus*, *P. aeruginosa*, *B. subtilis*	[65]
Lythraceae	*Punica granatum*	Antibacterial activity against *E. coli*, *S. aureus*, *S. epidermidis, P. aeruginosa*, *Proteus vulgaris*, *Salmonella typhi*, *K. pneumoniae*; Cytotoxicity against colon cancer cell line (RKO: ATCC^®^ CRL-2577™)	[66]
Bromeliaceae	*Ananas comosus*	Antioxidant activity; Cytotoxic effect against HepG2 (liver cancer cell line); Anti-diabetic activity; Antibacterial activity against *Bacillus cereus* KCTC 3624, *Listeria monocytogenes* ATCC 19111, *Enterococcus faecium* DB01, and *S. aureus* ATCC 13565	[67]
Sapindaceae	*Dimocarpus Longan Lour*	Antibacterial activity against *E. coli*, *S. aureus*, *P. aeruginosa*, *B. subtilis*; Antifungal activity against *Candida albicans*; Cytotoxicity against PC-3 (prostate cancer cell line)	[68]

**Table 6:** Applications of zinc oxide NPs synthesized from various fruit varieties.

Family	Fruit Verity	Applications	References
Rutaceae	*Citrus maxima*	Photocatalytic activity against methylene blue; Antibacterial activity against *Klebsiella aerogenes, E. coli*, *S. aureus*; Sensor activity towards dopamine	[69]
Clusiaceae	*Garcinia mangostana*	Photocatalytic activity against malachite green	[52]
Lythraceae	*Punica granatum*	Antibacterial activity against *Escherichia coli* and *Enterococcus faecalis*; Cytotoxicity against HCT116 (colorectal cancer cell line)	[70]
Bromeliaceae	*Ananas comosus*	Antibacterial activity against *E. coli*	[71]
